# Oral Health Knowledge, Attitudes, and Practices Among Male Primary School Students in Saudi Arabia: A School-Based Cross-Sectional Study

**DOI:** 10.3390/dj14080530

**Published:** 2026-08-19

**Authors:** Mohammed Khalid S. Alsharif, Ahmad Iqmer Nashriq Mohd Nazan, Ahmad Zaid Fattah Azman

**Affiliations:** 1 Public Health Department, College of Health Sciences at Al-Leith, Umm Al-Qura University, Makkah 24381, Saudi Arabia; mkshareef@uqu.edu.sa; 2 Department of Community Health, Faculty of Medicine and Health Sciences, Universiti Putra Malaysia, Serdang 43400, Selangor, Malaysia; ahmadzaid@upm.edu.my; 3 Public Health Section, Hospital Sultan Abdul Aziz Shah, Universiti Putra Malaysia, Serdang 43400, Selangor, Malaysia

**Keywords:** oral health, knowledge, attitudes, and practices, primary school children, Saudi Arabia, oral health literacy, dental caries

## Abstract

**Background/Objectives**: Dental caries remains one of the most prevalent and preventable childhood diseases worldwide. Despite universal access to free dental care in Saudi Arabia, preventable oral diseases remain common, highlighting persistent gaps in oral health literacy and preventive behaviours among children. Male students consistently demonstrate poorer oral health knowledge and practices than their female counterparts, yet they remain understudied. This study aimed to assess oral health knowledge, attitudes, and practices (KAP) among male primary school students in Al Lith city, Saudi Arabia, and to identify their associated sociodemographic determinants. **Methods**: A school-based analytical cross-sectional study was conducted during the 2023–2024 academic year in Al Lith city, Makkah Province, Saudi Arabia. A total of 430 male students aged 10–13 years from grades five and six were recruited via multistage cluster sampling from 8 randomly selected schools. A validated, interviewer-administered, Arabic-language questionnaire assessed knowledge (18 items), attitudes (12 Likert-scale items), and practices (12 items) related to oral health, alongside sociodemographic data. The study was designed and reported in accordance with the Strengthening the Reporting of Observational Studies in Epidemiology (STROBE) statement. Data were analysed using IBM SPSS Statistics version 27. Descriptive statistics and multivariable logistic regression were performed. **Results**: Of the 430 participants, 83.3% were aged 11–12 years, 89.8% were Saudi nationals, and 89.1% resided in intact, two-parent households. Knowledge deficits were widespread: only 8.4% correctly identified the recommended timing of sweet consumption, 20.2% understood dental plaque, and 28.6% recognised the protective role of fluoride. Attitudes were generally positive, with 80.1% agreeing that teeth should be brushed twice daily and 71.6% endorsing regular dental visits; however, 60.3% held that dental attendance was necessary only when pain was present. Substantial practice gaps were also evident: only 33.3% brushed for the recommended two minutes, and 12.6% had never visited a dentist. In multivariable analysis, low household income was independently associated with better oral health knowledge (adjusted odds ratio [AOR] = 2.22; 95% confidence interval [CI]: 1.04–4.74; *p* = 0.039), divorced parental status was independently associated with positive oral health attitudes (AOR = 5.40; 95% CI: 1.28–22.79; *p* = 0.022), and older age was independently associated with poorer oral health practices (AOR = 0.72; 95% CI: 0.55–0.93; *p* = 0.014). **Conclusions**: Male primary school students in Al Lith demonstrated generally positive oral health attitudes but significant deficits in knowledge and preventive practices. Comprehensive, school-based oral health promotion programmes that address key misconceptions, actively engage parents, and integrate culturally accepted practices—such as miswak use—with evidence-based preventive guidance are needed.

## 1. Introduction

Dental caries remains one of the most common yet preventable chronic diseases affecting children worldwide. According to the 2021 Global Burden of Disease Study, approximately 3.69 billion people worldwide are affected by oral conditions, with untreated dental caries in permanent teeth remaining the most prevalent condition assessed [1]. Recent evidence published in *The Lancet* identified oral diseases as a major global public health challenge, with childhood caries disproportionately affecting children in low- and middle-income settings and adversely affecting quality of life, school attendance, and academic performance [2]. In the Gulf Cooperation Council (GCC) region specifically, caries prevalence reaches 64.7% with a mean decayed, missing, and filled teeth (DMFT) of 2.57 [3]. Systematic review evidence further indicates that children with dental caries have substantially higher odds of poor school performance and absenteeism compared with caries-free peers [4], underscoring the broader educational consequences of this preventable condition. Despite the availability of free dental services, untreated dental caries continues to impose a substantial burden [5], suggesting that access to care alone is insufficient to improve oral health outcomes without strengthening oral health literacy and preventive behaviours. The World Health Organization (WHO) recognises oral health as integral to overall wellbeing, particularly during childhood when foundational oral hygiene habits are first established [6].

The Knowledge, Attitude, and Practice (KAP) framework is widely used to evaluate health-related behaviours. It encompasses awareness of preventive measures, affective responses to oral health, and the adoption of preventive behaviours. Evidence suggests that knowledge alone is unlikely to translate into appropriate oral health behaviours; attitudes also play an important role in shaping preventive practices [6,7].

In Saudi Arabia, despite universal provision of free dental care, oral health remains a significant public health concern [3]. The burden of oral diseases is strongly influenced by social determinants, including income, education, and access to healthcare, which contribute to persistent oral health inequalities [7]. Gender is a significant determinant of oral health behaviour: global evidence consistently demonstrates that females exhibit better oral hygiene practices and higher toothbrushing frequency than males [8], with a large-scale retrospective review of over 9000 Japanese university freshmen confirming that males brush less frequently and for shorter durations than females [9]. In Saudi Arabia specifically, males present with higher plaque and gingival index scores, indicative of poorer oral hygiene [10]. These gender-specific disparities underscore the need for targeted oral health promotion strategies addressing the unique oral health needs of male students.

Sociodemographic factors—including parental education, family income, and socioeconomic status—also significantly influence children’s oral health outcomes. A large cross-sectional study of 8446 Chinese families demonstrated a complex, non-linear relationship between parental education and children’s oral health behaviours, with higher parental education not consistently predicting better child oral health practices [11]. Schools represent ideal settings for oral health promotion, affording access to large child populations during critical developmental stages; school-based oral health education programmes in Saudi Arabia have demonstrated improvements in children’s knowledge, attitudes, practices, and clinical outcomes [12].

Although KAP research on oral health is abundant, studies focusing specifically on male primary school students in Saudi Arabia remain scarce. Given documented gender differences and limited male-specific data, this study aimed to assess oral health KAP among male primary school students in Al Lith, Saudi Arabia, and to identify associated sociodemographic determinants, with the overarching goal of informing targeted, school-based oral health promotion programmes.

## 2. Materials and Methods

### 2.1. Research Design and Reporting Standards

A school-based analytical cross-sectional study was undertaken to assess oral health knowledge, attitudes, and practices (KAP) among male primary school students in Al Lith, Saudi Arabia. The study was designed and reported in accordance with the Strengthening the Reporting of Observational Studies in Epidemiology (STROBE) statement [13].

### 2.2. Study Setting

The study was carried out in Al Lith city, located in the Tihamah coastal region of Makkah Province, Saudi Arabia, approximately 180 km southwest of Makkah and 190 km south of Jeddah. Eligible male primary schools in Al Lith city constituted the sampling frame. Data collection was conducted during the 2023–2024 academic year. A school-based setting was chosen because it enabled efficient access to the target population, promoted standardised data collection procedures, and reduced potential selection bias compared with community-based recruitment.

### 2.3. Study Population and Eligibility

The target population comprised male students aged 10–13 years enrolled in grades five and six. Inclusion criteria were as follows: male sex; age 10–13 years; enrolment in a selected school during the study period; and written informed parental or guardian consent. Students were excluded if they were medically unfit at the time of data collection, had cognitive or communication limitations precluding reliable questionnaire completion, or were absent during the scheduled data collection period.

### 2.4. Sample Size Determination

The sample size was calculated using a cluster randomised trial formula based on a previous school-based oral health intervention study with comparable outcome measures [14]. The calculation assumed 80% statistical power, a 95% confidence level, and proportions of adequate oral health knowledge of 90.3% in the intervention group and 63.6% in the control group. A design effect of 2.2 was applied to account for clustering, and the sample size was increased by 20% to allow for non-response or incomplete data. The required sample size was 215 participants per group, yielding a total target sample of 430 students.

### 2.5. Sampling Strategy

A multistage cluster sampling approach was employed. In the first stage, eight eligible male primary schools were randomly selected from all eligible male primary schools in Al Lith city using simple random sampling. In the second stage, eligible students from grades five and six were randomly selected from school enrolment records until the required sample size of 430 participants was achieved. Participants were proportionally allocated across the selected schools to ensure adequate representation of the target population.

### 2.6. Data Collection Procedure

A team of trained data collectors administered the interviewer-administered Arabic questionnaire through face-to-face interviews in classroom settings, following coordination with school administrations. Prior to fieldwork, all data collectors received standardised training covering study procedures, ethical considerations, and questionnaire administration to ensure consistency and minimise interviewer bias. The study purpose was explained in clear, age-appropriate language; participation was voluntary, and confidentiality was assured. Each interview session lasted approximately 25–30 min. Completed questionnaires were reviewed on-site for completeness, and all data were anonymised and stored securely.

### 2.7. Study Instrument

The interviewer-administered Arabic-language questionnaire was developed by adapting previously published and validated oral health questionnaires, guided by the WHO Oral Health Surveys: Basic Methods (5th edition), with minor adaptations to ensure age appropriateness and cultural relevance for Saudi primary school children [15,16,17]. The questionnaire was translated from English into Arabic using a forward–backward translation process, and the Arabic version was reviewed by two bilingual language experts to ensure linguistic accuracy and conceptual equivalence. A pilot study involving 30 male primary school students from a school outside the study sample was conducted to evaluate the clarity, comprehensibility, and suitability of the questionnaire, and minor revisions were made before the main study. Face validity was assessed among male primary school students outside the study sample, and the Arabic version was further reviewed by four Arabic language supervisors from the Ministry of Education to ensure clarity, readability, age appropriateness, and cultural suitability. Content validity was evaluated by experts in community health, health promotion, and epidemiology, and their recommendations were incorporated into the final version of the questionnaire.

The questionnaire comprised four domains: (1) Sociodemographic information: Age, grade level, parental education, parental occupation, household income (monthly income categories in Saudi Arabian Riyal (SAR)), and nationality. (2) Knowledge domain: 18 multiple-choice questions covering dental anatomy, dental caries, gingivitis, and oral cancer. Each correct response was scored as 1 and each incorrect response as 0, yielding a total score ranging from 0 to 18. (3) Attitude domain: 12 items rated on a 5-point Likert scale ranging from 1 (strongly disagree) to 5 (strongly agree). Negatively worded items were reverse-scored to maintain directional consistency. For the binary logistic regression analyses, the attitude items were dichotomously recoded as favourable (1) or unfavourable (0), and the recoded items were summed to generate an analytical attitude score ranging from 0 to 12. (4) Practice domain: 12 multiple-choice questions assessing oral health practices, including toothbrushing frequency and duration, toothbrush type, interdental cleaning, and dental attendance patterns. Each practice item was dichotomously scored, with 1 assigned to an appropriate oral health practice and 0 to an inappropriate oral health practice, yielding a total practice score ranging from 0 to 12, with higher scores indicating better oral health practices. The internal consistency of the knowledge, attitude, and practice domains was assessed using Cronbach’s alpha. The coefficients were 0.78 for knowledge, 0.81 for attitude, and 0.76 for practice, indicating acceptable-to-good internal consistency across all three domains. As no validated or standardised cut-off values were available for the knowledge, attitude, and practice outcomes, study-specific operational thresholds were used for categorising participants. The sample mean was used as the operational threshold for the knowledge and attitude analytical scores, whereas a score of 6, corresponding to 50% of the maximum possible practice score, was used for the practice outcome. Participants scoring below the respective thresholds were classified as having poor knowledge, negative attitudes, or poor oral health practices, whereas those scoring at or above the thresholds were classified as having good knowledge, positive attitudes, or good oral health practices. Accordingly, knowledge scores were categorised as poor (<6.16) or good (≥6.16), attitude scores as negative (<6.87) or positive (≥6.87), and practice scores as poor (<6) or good (≥6). The English version of the questionnaire is provided as Supplementary Material (Supplementary File S1).

### 2.8. Statistical Analysis

Data were entered, cleaned, and analysed using IBM SPSS Statistics version 27 (IBM Corp., Armonk, NY, USA). Descriptive statistics were used to summarise participants’ sociodemographic characteristics and oral health knowledge, attitudes, and practices. Categorical variables were presented as frequencies and percentages. The KAP outcomes were dichotomised using the study-specific operational thresholds described in Section 2.7. The sociodemographic variables included in the models were nationality, parental marital status, parental employment, household income, and age. Multivariable binary logistic regression was performed at the individual participant level to identify independent sociodemographic predictors of KAP outcomes. Clustering of students within schools was not accounted for in the regression models. Adjusted odds ratios (AORs), 95% confidence intervals (CIs), and *p*-values were reported. All tests were two-tailed, and statistical significance was set at *p* < 0.05.

### 2.9. Ethical Considerations

Ethical approval was obtained from the Ethics Committee for Research Involving Human Subjects (JKEUPM), Universiti Putra Malaysia (Reference No. JKEUPM-2023-634; Approval Date: 25 October 2023), and the Ministry of Education in Saudi Arabia (Reference No. 4401270322; Approval Date: 7 July 2023) prior to data collection. Written informed consent was obtained from the parents or legal guardians of all participating students, and assent was obtained from the students before participation. Participation was entirely voluntary, and participants were informed of their right to withdraw at any stage without consequence. No personally identifiable information was collected; all data were anonymised, stored securely, and used solely for research purposes. All procedures were conducted in accordance with the Declaration of Helsinki.

## 3. Results

### 3.1. Sociodemographic Characteristics

A total of 430 male schoolchildren were enrolled (Table 1). Students were distributed between fifth grade (55.6%) and sixth grade (44.4%). The majority (83.3%) were aged 11–12 years. Most were Saudi nationals (89.8%), lived with parents or guardians (97.7%), and came from intact, married-parent households (89.1%).

Parental socioeconomic profiles indicated a relatively advantaged population. Most fathers (57.2%) were employed full-time, and 43.5% held undergraduate qualifications or higher. Over half of mothers were housewives (54.7%), while 52.1% held undergraduate qualifications or higher. More than half of the households reported middle-range incomes (54.0%).

### 3.2. Oral Health Knowledge

Knowledge levels were heterogeneous, revealing substantial misconceptions across all assessed domains (Table 2). Regarding dentition, participants showed acceptable recognition of the most prevalent childhood dental disease (71.6%); however, basic anatomical knowledge was poor: only 20.2% correctly identified the number of teeth in a child’s mouth, and only 8.4% identified the recommended timing of sweet consumption—indicating a critical gap in understanding cariogenic dietary patterns. Only 32.8% recognized the importance of primary (deciduous) teeth.

Knowledge related to dental caries was generally limited. While 52.8% correctly defined tooth decay, fewer understood its causes (41.4%), preventive measures (34.2%), or the protective role of fluoride (28.6%).

Knowledge of gingivitis and periodontal health was limited. While 61.4% recognized bleeding during toothbrushing as a warning sign, only 20.2% understood the concept of dental plaque, and 20.9% knew how gum disease could be prevented. Knowledge of oral cancer was weakest overall: only 26.5% could define oral cancer, 32.1% correctly identified its causes, and 35.8% knew preventive approaches.

### 3.3. Attitudes Toward Oral Health

Attitudinal responses revealed a generally positive orientation toward oral hygiene, although important misconceptions persisted (Table 3). Most participants agreed that toothbrushing and flossing reduce tooth decay (71.6%), that teeth should be brushed at least twice daily (80.1%), that teeth and gums require daily cleaning (81.7%), and that regular dental visits are necessary (71.6%).

However, misconceptions were substantial. Nearly half (45.4%) agreed that only dentists can prevent cavities, reflecting low perceived self-efficacy for home-based prevention. More than three in five (60.3%) agreed that dental visits are necessary only when pain is present, reflecting a symptom-driven rather than preventive orientation. Additionally, 53.5% believed that flossing should cease when gums bleed, indicating a misunderstanding of gingival inflammation. Approximately one-third believed that tooth loss before the age of five is normal, further underscoring the undervaluation of primary dentition [18].

### 3.4. Oral Health Practices

Reported practices revealed a clear knowledge–behaviour gap (Table 4). Although 58.1% brushed twice daily, 21.4% brushed only once daily and 20.4% less frequently. Toothpaste use was near universal (95.1%), but only 53.5% knowingly used fluoridated toothpaste, while 38.8% were uncertain of the fluoride content of their toothpaste. Brushing duration was suboptimal: only 33.3% reported brushing for the recommended two minutes, and over half brushed for one minute or less.

Interdental hygiene practices were inconsistent. Although 51.9% reported cleaning between teeth daily or more frequently, the use of wooden (59.1%) and plastic toothpicks (29.5%) was common among participants. Miswak use was highly prevalent (83.5%), reflecting deeply rooted cultural oral hygiene traditions.

Toothbrush maintenance was mixed: 44.2% replaced their brush every 60–90 days, but 19.5% were unsure when replacement should occur. Only 44.0% used soft-bristled brushes. Dental attendance was moderate: 41.2% attended regularly, 34.7% irregularly, and 12.6% had never visited a dentist.

### 3.5. Multivariable Predictors of KAP Outcomes

Table 5 presents adjusted odds ratios (AORs) from multivariable logistic regression models examining factors associated with oral health knowledge, attitudes, and practices. After adjustment for all covariates, only a limited number of sociodemographic factors remained independently associated with KAP outcomes.

For knowledge, household income was the sole independent predictor. Participants from low-income households had more than twice the odds of demonstrating good oral health knowledge relative to those from high-income households (AOR = 2.22; 95% CI: 1.04–4.74; *p* = 0.039).

For attitudes, family structure was independently associated with outcomes. Children whose parents were divorced demonstrated substantially higher odds of holding positive attitudes compared with those whose parents were widowed (AOR = 5.40; 95% CI: 1.28–22.79; *p* = 0.022). Additionally, children whose mothers were housewives had higher odds of positive attitudes relative to those with retired mothers (AOR = 3.15; 95% CI: 1.03–9.64; *p* = 0.045).

For practices, age was the only independent predictor: each additional year of age was associated with lower odds of reporting good oral health practices (AOR = 0.72; 95% CI: 0.55–0.93; *p* = 0.014).

## 4. Discussion

This study assessed oral health KAP among male primary school students in Al Lith, Saudi Arabia, revealing substantial gaps in oral health knowledge and practice despite generally positive attitudinal profiles. The mismatch between positive attitudes and limited knowledge and practices is consistent with the KAP framework, which recognises that knowledge deficits, low self-efficacy, and environmental barriers collectively impede translation of positive attitudes into sustained preventive behaviour [6,12]. These findings should be interpreted in the context of the global oral disease burden: the 2021 GBD analysis confirmed that oral conditions collectively affect 3.69 billion people, with untreated caries in permanent teeth ranking as the most prevalent condition globally [1]; *The Lancet* Oral Health Series characterised oral diseases as a major public health challenge warranting radical action [2,19].

Most participants were aged 11–12 years, a developmental stage during which lifelong health habits are consolidated. The WHO identifies school-aged children as a priority population for oral health promotion, given that behaviours established during childhood tend to persist into adulthood [6]. Despite the relatively favourable socioeconomic profile of the participants, including generally stable family environments and relatively high parental educational attainment, substantial deficits in oral health knowledge and preventive practices were observed. This is consistent with large-scale evidence from China demonstrating that parental education level does not consistently translate into improved child oral health knowledge or practice [11], and with findings from the United Arab Emirates and Saudi Arabia where similar patterns have been observed [20,21].

Knowledge deficits were pervasive across all domains. Only 8.4% correctly identified the appropriate timing of sweet consumption, which is of clinical importance given the well-established relationship between frequent free-sugar exposure and enamel acid demineralisation. The WHO recommends restricting free-sugar intake to below 10% of total energy and reducing sugar exposure frequency to minimise caries risk [6]. The systematic review by Moynihan and Kelly [22] demonstrated that higher sugar intake is directly associated with greater caries incidence across the life course, and the 2022 ten-year update by Moores, Kelly, and Moynihan [23] extended this evidence base to 2020, confirming that restriction of sugar intake remains a cornerstone of caries prevention. The present study’s findings therefore point to a critical gap in dietary oral health education that must be addressed in future programmes.

Fluoride literacy was particularly low, with fewer than one third of participants recognising fluoride’s protective role in caries prevention. This finding is particularly concerning given that dental caries in permanent teeth remains the most prevalent condition identified in the 2021 Global Burden of Disease oral health analysis [1]. Fluoride toothpaste is among the most cost-effective and evidence-based caries prevention interventions available; updated Cochrane systematic review evidence confirms its efficacy across a wide range of concentrations [24]. The combination of low fluoride knowledge and high rates of uncertainty regarding the fluoride content of the toothpaste used indicates a clear need for explicit preventive guidance on toothpaste selection. This finding is consistent with global oral health literacy deficits documented across GCC countries [3].

Awareness of the importance of primary teeth was also limited, with fewer than one third of participants recognising their value. Primary teeth play a fundamental role in mastication, speech development, craniofacial growth, nutrition, and maintaining space for permanent teeth. A systematic review emphasised that parents globally require greater awareness of the importance of deciduous teeth and the need for regular dental attendance for their children [18]. Oral cancer knowledge was the weakest domain assessed. Although certain concepts related to oral cancer, such as human papillomavirus (HPV), may be relatively advanced for primary school children, the questionnaire was intended to assess general awareness of oral cancer and its prevention rather than detailed or specialist knowledge. Despite the rarity of oral cancer in childhood, early tobacco-prevention messaging carries long-term public health value because risk behaviours initiated in youth frequently persist into adulthood.

Attitudinal profiles were generally positive, consistent with studies from Saudi Arabia [20] and the UAE [21]. These favourable attitudes may provide an important foundation for future school-based behaviour-change interventions. Nevertheless, 45.4% of participants believed that only dentists could prevent dental caries, suggesting limited preventive self-efficacy. Furthermore, 60.3% held that dental attendance is warranted only when pain occurs, reflecting a predominantly treatment-seeking rather than preventive orientation. Similar symptom-driven patterns of dental attendance have been reported among Saudi schoolchildren, where dental pain was the most common reason for seeking dental care, whereas routine preventive dental visits were substantially less frequent [25]. The belief that flossing should cease when gums bleed further illustrates a misunderstanding of gingival inflammation, which is particularly relevant given that interprofessional guidance consistently advocates continued interdental cleaning in the presence of gingivitis as an essential therapeutic measure [26].

Practice findings demonstrated a clear knowledge–behaviour gap consistent with the KAP framework [12]. Although toothpaste use was near universal, only approximately one third of participants brushed for the recommended two minutes, and only 53.5% knowingly used fluoridated toothpaste—a finding that, combined with the low fluoride knowledge documented above, represents a significant missed prevention opportunity. Only 44.0% of participants reported using soft-bristled toothbrushes, while the remainder either used other bristle types or were unsure of the type used. This finding suggests limited awareness regarding appropriate toothbrush selection and highlights an additional area for oral health education. The widespread use of wooden and plastic toothpicks rather than evidence-based interdental cleaning tools warrants attention, as improper toothpick use may increase the risk of gingival trauma. Furthermore, meta-review evidence supports the superiority of dental floss and interdental brushes over toothpicks for plaque removal and gingivitis control [26].

One culturally distinctive finding was the very high prevalence of miswak use (83.5%). Accumulating evidence, including a recent systematic review of 10 randomised controlled trials, confirms miswak’s antimicrobial, antibiofilm, anti-inflammatory, and plaque-reducing properties [27]. Given its deep cultural significance in Muslim-majority communities, oral health promotion programmes should thoughtfully integrate miswak within evidence-based preventive frameworks, rather than treating traditional practices as incompatible with contemporary recommendations.

Multivariable analysis revealed that sociodemographic factors accounted for only a limited proportion of KAP variance, consistent with broader evidence that oral health behaviours are primarily shaped by psychosocial, educational, and cultural influences rather than demographic characteristics alone [7]. Unexpectedly, lower household income was associated with better oral health knowledge. This association may reflect greater reliance on school-based and public health education among lower-income households, whereas higher-income households may have greater access to private dental services and therefore rely less on population-level oral health education programmes. However, this explanation remains speculative. The finding should also be interpreted cautiously because of possible residual confounding, the relatively wide confidence interval, and the cross-sectional nature of the study. Replication in larger and more diverse populations is warranted. Similarly, the association between divorced parental status and positive oral health attitudes should be interpreted cautiously because of the wide confidence interval, which indicates considerable uncertainty in the estimate. This imprecision may largely reflect the small size of the widowed reference group, together with the relatively small number of participants in the divorced parental-status category. Despite these limitations, the observed associations underscore the potential value of school-based health promotion in reaching socially vulnerable populations, consistent with the equity-based arguments advanced in the 2019 Lancet Oral Health Series [2,19].

The WHO Health Promoting Schools framework provides a Cochrane-evaluated mechanism for integrating oral health into school systems through curriculum-based education, supervised toothbrushing, healthy food policies, and regular dental screening [28]. School-based oral health education programmes in Saudi Arabia have demonstrated measurable improvements in KAP outcomes [12], suggesting the feasibility and acceptability of this approach. Targeted interventions addressing the specific misconceptions identified in this study—regarding fluoride use, sweet consumption timing, management of bleeding gums, the role of preventive dental visits, and the importance of primary dentition—are needed. Parental engagement is equally essential, as home supervision directly influences brushing behaviour, dietary choices, and healthcare utilisation [11,18]. A school-based approach in Al Lith that incorporates structured parent education components, culturally affirming miswak integration, and fluoride literacy modules would respond directly to the deficits identified herein. These efforts should be informed by the equity-centred frameworks advocated in the global oral health literature [7,19] and evaluated within the WHO Health Promoting Schools model [28].

### Strengths and Limitations

This study has several notable strengths. It included a relatively large, systematically sampled cohort of male primary schoolchildren and employed a multidimensional KAP assessment alongside sociodemographic variables, enabling a broader understanding of behavioural determinants than studies addressing a single domain in isolation. The use of trained data collectors and a standardised interviewer-administered questionnaire likely improved response completeness and reduced misunderstanding of questionnaire items among younger participants. Multivariable regression allowed identification of independent predictors with adjustment for potential confounders, and STROBE-compliant reporting [13] enhances transparency and reproducibility.

However, several limitations warrant acknowledgement. First, the cross-sectional design precludes causal inference; longitudinal and interventional studies are needed to elucidate causal pathways and evaluate long-term outcomes. Second, self-reported behavioural practices are susceptible to recall and social desirability bias, particularly because the participants were children, potentially overestimating behaviours such as toothbrushing frequency and duration. Third, the study was conducted in a single city, limiting generalisability to other regions with different socioeconomic or cultural profiles. Fourth, clinical oral examinations were not performed; consequently, reported knowledge and practices could not be compared with objective oral health indicators, such as dental caries, plaque accumulation, or gingival inflammation. Fifth, household income was self-reported by the participating children using predefined income categories rather than exact income values. Although uncertain responses were clarified with assistance from the class teacher during data collection, some degree of reporting inaccuracy cannot be completely excluded, particularly among younger participants. Sixth, the multivariable logistic regression models did not account for the clustering of students within schools. Consequently, the standard errors may have been underestimated, and the confidence intervals may have been narrower than those obtained from a cluster-adjusted analysis. The regression findings should therefore be interpreted with caution. Finally, the study included only male students from one city; therefore, the findings cannot be generalised to female students or to all Saudi Arabian schoolchildren.

## 5. Conclusions

Male primary school students in Al Lith, Saudi Arabia, demonstrated generally positive attitudes toward oral health but substantial gaps in oral health knowledge and preventive practices. Comprehensive school-based oral health promotion programmes, supported by active parental engagement and culturally sensitive approaches that integrate evidence-based guidance with traditional practices such as miswak use, are needed to improve oral health literacy and promote healthier oral health behaviours. Future longitudinal and interventional studies incorporating clinical oral examinations are warranted to evaluate the long-term effectiveness of preventive strategies.

## Figures and Tables

**Table 1:** Sociodemographic Characteristics of Participants (*N* = 430).

Variable	Category	*n*	%
Class Level	Fifth Grade	239	55.6
	Sixth Grade	191	44.4
Age (years)	10	50	11.6
	11	184	42.8
	12	174	40.5
	13	22	5.1
Nationality	Saudi	386	89.8
	Non-Saudi	44	10.2
Living Arrangement	With parent(s)	420	97.7
	Without parent(s)	10	2.3
Parental Marital Status	Married	383	89.1
	Divorced	32	7.4
	Widowed	15	3.5
Father’s Employment	No employment	49	11.4
	Self-employed	28	6.5
	Part-time	40	9.3
	Full-time	246	57.2
	Retired	67	15.6
Mother’s Employment	Housewife	235	54.7
	Self-employed	11	2.6
	Part-time	30	7.0
	Full-time	138	32.1
	Retired	16	3.7
Father’s Education	Illiterate	26	6.0
	Intermediate or lower	77	17.9
	High school	140	32.6
	Undergraduate or higher	187	43.5
Mother’s Education	Illiterate	29	6.7
	Intermediate or lower	71	16.5
	High school	106	24.7
	Undergraduate or higher	224	52.1
Household Income (SAR) *	<5000 (Low)	120	27.9
	5000–14,999 (Middle)	232	54.0
	≥15,000 (High)	78	18.1

* SAR, Saudi Arabian Riyal.

**Table 2:** Oral Health Knowledge Among Participants (*N* = 430).

Question	Correct *n* (%)	Incorrect *n* (%)
**Dentition**		
How many teeth are in a child’s mouth?	87 (20.2)	343 (79.8)
Which factors can lead to crooked teeth?	92 (21.4)	338 (78.6)
What is the most common dental disease in children?	308 (71.6)	122 (28.4)
What is the purpose of brushing teeth?	188 (43.7)	242 (56.3)
When is the appropriate time to eat sweets?	36 (8.4)	394 (91.6)
What is the importance of primary (deciduous) teeth?	141 (32.8)	289 (67.2)
**Dental Caries**		
What is tooth decay?	227 (52.8)	203 (47.2)
What causes tooth decay?	178 (41.4)	252 (58.6)
Which factors prevent tooth decay?	147 (34.2)	283 (65.8)
What are the effects of fluoride on teeth?	123 (28.6)	307 (71.4)
Which foods can cause tooth decay?	147 (34.2)	283 (65.8)
**Gingivitis**		
What is dental plaque?	87 (20.2)	343 (79.8)
Blood on a toothbrush may indicate?	264 (61.4)	166 (38.6)
What causes bleeding gums?	126 (29.3)	304 (70.7)
How can gum disease be prevented?	90 (20.9)	340 (79.1)
**Oral Cancer**		
What is oral cancer?	114 (26.5)	316 (73.5)
What can cause oral cancer?	138 (32.1)	292 (67.9)
How can oral cancer be prevented?	154 (35.8)	276 (64.2)

**Table 3:** Attitudes Toward Oral Health Among Participants (*N* = 430).

Statement	Strongly Disagree *n* (%)	Disagree *n* (%)	Neutral *n* (%)	Agree *n* (%)	Strongly Agree *n* (%)
Only the dentist can prevent cavities	99 (23.0)	121 (28.1)	105 (24.4)	72 (16.7)	33 (7.7)
Toothbrushing and flossing reduces tooth decay	35 (8.1)	70 (16.3)	18 (4.2)	106 (24.7)	201 (46.7)
Tooth loss before age 5 is normal	143 (33.3)	144 (33.5)	9 (2.1)	99 (23.0)	35 (8.1)
I am responsible for preventing tooth loss	74 (17.2)	59 (13.7)	21 (4.9)	130 (30.2)	146 (34.0)
Teeth should be brushed at least twice daily	39 (9.1)	41 (9.5)	5 (1.2)	88 (20.5)	257 (59.8)
Flossing should stop if gums bleed	75 (17.4)	88 (20.5)	67 (15.6)	140 (32.6)	60 (14.0)
Dental visits are necessary only when in pain	77 (17.9)	92 (21.4)	20 (4.7)	141 (32.8)	100 (23.3)
Teeth and gums need cleaning every day	32 (7.4)	39 (9.1)	10 (2.3)	96 (22.3)	253 (58.8)
Fluoridated toothpaste is beneficial for teeth	31 (7.2)	33 (7.7)	71 (16.5)	150 (34.9)	145 (33.7)
Sugary foods and drinks should be avoided	60 (14.0)	54 (12.6)	25 (5.8)	139 (32.3)	152 (35.3)
Primary (deciduous) teeth should be treated	51 (11.9)	55 (12.8)	63 (14.7)	136 (31.6)	125 (29.1)
Regular dental check-ups are necessary	46 (10.7)	39 (9.1)	22 (5.1)	129 (30.0)	194 (45.1)

**Table 4:** Oral Health Practices Among Participants (*N* = 430).

Variable	Category	*n*	%
Parental assistance with brushing	Yes	162	37.7
	No	268	62.3
Brushing frequency	Twice a day	250	58.1
	Once a day	92	21.4
	2–3 times per week	50	11.6
	Less frequently	38	8.8
Toothpaste use	Yes	409	95.1
	No	5	1.2
	Never brushes	12	2.8
	Unsure	4	0.9
Fluoridated toothpaste use	Yes	230	53.5
	No	33	7.7
	Unsure	167	38.8
Brushing duration	<1 min	72	16.7
	1 min	152	35.3
	2 min	143	33.3
	Unsure	63	14.7
Interdental cleaning frequency	Once daily or more	223	51.9
	Once weekly	37	8.6
	Less frequently	170	39.5
Toothbrush replacement	Every 60–90 days	190	44.2
	Every 6 months	81	18.8
	Every 6–12 months	10	2.3
	Once yearly	26	6.0
	When bristles wear out	39	9.1
	Unsure	84	19.5
Toothbrush bristle hardness	Hard	37	8.6
	Soft	189	44.0
	Medium	121	28.1
	Does not matter	10	2.3
	Unsure	73	17.0
Wooden toothpick use	Yes	254	59.1
	No	148	34.4
	Unsure	28	6.5
Plastic toothpick use	Yes	127	29.5
	No	262	60.9
	Unsure	41	9.5
Miswak (chewstick) use	Yes	359	83.5
	No	57	13.3
	Unsure	14	3.3
Dental attendance	Regular check-up	177	41.2
	Irregular attendance	149	34.7
	Never attended	54	12.6
	Cannot recall	50	11.6

**Table 5:** Multivariable Logistic Regression: Sociodemographic Predictors of Oral Health Knowledge, Attitude, and Practice Outcomes.

Variable	Category	Knowledge, AOR (95% CI)	*p*	Attitude, AOR (95% CI)	*p*	Practice, AOR (95% CI)	*p*
Nationality	Saudi vs. non-Saudi	0.74 (0.36–1.55)	0.431	1.18 (0.57–2.45)	0.657	1.35 (0.65–2.82)	0.419
Parental marital status	Married vs. Widowed	5.78 (0.70–47.89)	0.104	1.99 (0.62–6.35)	0.246	0.99 (0.29–3.36)	0.991
	Divorced vs. Widowed	4.94 (0.53–45.68)	0.159	5.40 (1.28–22.79)	0.022 *	1.10 (0.28–4.38)	0.892
Father’s employment	No employment vs. Retired	0.43 (0.17–1.05)	0.064	0.92 (0.40–2.14)	0.853	0.88 (0.38–2.04)	0.764
	Self-employed vs. Retired	0.44 (0.15–1.25)	0.122	1.43 (0.53–3.84)	0.478	0.54 (0.19–1.49)	0.235
	Part-time vs. Retired	1.27 (0.51–3.12)	0.606	2.08 (0.83–5.20)	0.118	1.54 (0.64–3.75)	0.338
	Full-time vs. Retired	0.80 (0.42–1.53)	0.497	1.53 (0.81–2.90)	0.186	1.07 (0.57–1.99)	0.841
Mother’s employment	Housewife vs. Retired	1.49 (0.45–4.87)	0.514	3.15 (1.03–9.64)	0.045 *	0.42 (0.14–1.32)	0.138
	Self-employed vs. Retired	1.44 (0.26–7.95)	0.674	2.83 (0.53–15.13)	0.225	1.21 (0.21–6.86)	0.830
	Part-time vs. Retired	0.58 (0.13–2.56)	0.468	2.01 (0.52–7.79)	0.311	0.25 (0.06–1.01)	0.051
	Full-time vs. Retired	0.95 (0.28–3.26)	0.932	2.44 (0.77–7.72)	0.129	0.40 (0.12–1.28)	0.121
Household income	Low vs. High	2.22 (1.04–4.74)	0.039 *	1.05 (0.50–2.18)	0.904	1.33 (0.64–2.75)	0.441
	Middle vs. High	1.27 (0.69–2.31)	0.439	0.97 (0.55–1.71)	0.918	0.65 (0.37–1.12)	0.119
Age (per year)	—	0.82 (0.62–1.08)	0.160	1.26 (0.96–1.66)	0.101	0.72 (0.55–0.93)	0.014 *

AOR, adjusted odds ratio; CI, confidence interval. * *p* < 0.05.

## Data Availability

The data presented in this study are available on reasonable request from the corresponding author. The data are not publicly available due to ethical and privacy restrictions.

## Supplementary Materials

The following supporting information can be downloaded at: https://www.mdpi.com/article/10.3390/dj14080530/s1, Supplementary File S1: Oral Health Knowledge, Attitudes, and Practices Questionnaire.

## Abbreviations

The following abbreviations are used in this manuscript:

AOR	Adjusted Odds Ratio
CI	Confidence Interval
DMFT	Decayed, Missing, and Filled Teeth
GBD	Global Burden of Disease
GCC	Gulf Cooperation Council
HPV	Human Papillomavirus
KAP	Knowledge, Attitude, and Practice
SAR	Saudi Arabian Riyal
WHO	World Health Organization
