# Supplementary material for: Oral Health Knowledge, Attitudes, and Practices Among Male Primary School Students in Saudi Arabia: A School-Based Cross-Sectional Study
Source: Dent J (Basel). 2026 Aug 19;14(8):530. doi: 10.3390/dj14080530 (PMC13510636; doi:10.3390/dj14080530)

Questionnaire

IDENTIFICATION NO. | | | | | | |

EFFECTIVENESS OF SOCIAL COGNITIVE THEORY-BASED ORAL HEALTH EDUCATION ON KNOWLEDGE, ATTITUDE, AND PRACTICE AMONG MALE PRIMARY SCHOOLCHILDREN IN AL-LITH

Grade: ____________________________________

Please read the following questions carefully and answer them truthfully, you can ask for clarification.

This questionnaire will remain confidential and used for research purposes only.

Section A: Socio-demographic characteristics

1. **What is your age (in years)?_________**(years)
2. **What is your nationality?**

Saudi	Non-Saudi

3. **What is your grade?**

Grade 5	Grade 6

4. **Who do you live with? (Tick all that applies)**

Mother and father	Grandparents	Uncle/aunt (father's side)
Uncle/aunt (mother's side)	Siblings	Others (Please specify):

5. **What is the marital status of your parents?**

Married	Separated	Divorced	Both deceased (Mother & Father)
Mother deceased	Father deceased	Other (please specify):

6. **What is your father's occupation?**

Full-time work	Part-time work	Self-employed
Retired	Not employed

7. **What is your mother's occupation?**

Full-time work	Part-time work	Self-employed
Retired	Housewife

8. **What is your father's education?**

Cannot read or write	Intermediate school or below	High school
Undergraduate degree or higher.

9. **What is your mother's education?**

Cannot read or write	Intermediate school or below	High school
Undergraduate degree or higher.

10. **What is your family's monthly income?**

< 5000 SR (Low income)	5000–14,999 SR (Middle income)	≥ 15,000 SR (High income)

Section B: Knowledge of oral health

Please tick one answer only (√) as appropriate for each part

A. Knowledge of dentition (K-Dent, score: 0– 6)

1. How many teeth are there in a child's mouth?

a. 10 b. 12 c. 20 d. 28 e. I don't know

2. Which of the following can lead to irregular teeth?

a. Thumb sucking b. Tongue thrusting c. Mouth breathing
d. All of the above I don't know

3. What is the most common dental disease among children?

a. Tooth decay b. Bleeding gums c. Discoloration of teeth d. I don't know

4. What is the purpose of brushing your teeth?

a. To remove bacteria (germs) from all tooth surfaces b. To remove food from tooth surfaces
c. Both a and b (to remove germs, to remove food) d. I don't know

5. When is the most "tooth-friendly" time to have a sugary treat?

a. First thing in the morning b. Last thing at night c. Along with a meal
d. As a snack on its own e. It doesn't make any difference f. I don't know

6. What is the importance of milk (baby) teeth?

a. It is not important; they are temporary teeth b. It is important if there is a toothache only
c. It is important to keep them in a good condition and prevent them from diseases d. I don't know

B. Knowledge of dental caries (K-Caries, score:0–5)

7. What is tooth decay?

a. Damage to a tooth's surface, or enamel b. Gum bleeding
c. A layer of germs on the teeth d. I don't know

8. What causes tooth decay?

a. Too much consumption of sugary food and candies b. Unclean teeth
c. All of the above d. I don't know

9. Which of the following helps prevent tooth decay?

a. Tooth brushing b. Using fluoridated toothpaste c. Reducing the frequency of sugar consumption
d. Visiting the dentist regularly e. All of the above f. I don't know

10. What is the effect of fluoride on the teeth?

a. No effect b. Prevents tooth decay
c. Whitens teeth d. Helps prevent gum disease
e. I don't know

11. Which of the following foods can cause tooth decay?

a. Soft drinks b. Ice-cream c. Bakery products
d. Potato chips e. Chocolate f. All of the above g. I don't know

C. Knowledge of gingivitis (K-Gingivitis, score: 0–4)

12. What is plaque?

a. A toothpaste
b. A layer of germs on the teeth
c. A plastic coating for teeth
d. I don't know

13. Blood on your toothbrush may be a sign of:

a. Plaque.
b. Gum disease (gingivitis).
c. Tooth decay
d. I don't know

14. What are the causes of gum bleeding?

a. Not cleaning teeth
b. Improper brushing
c. All of the above
d. I don’t know

15. How can periodontal disease (gum disease) be prevented?

a. Regular tooth brushing
b. Saline mouth-rinsing
c. Regular scaling (professional tooth cleaning)
d. All of the above
I don’t know

D. Knowledge of causes of oral cancer (K-Cancer, score: 0–3)

16. What is oral cancer?

a. Malignant growth or tumour in tissues of the oral cavity (the mouth)
b. Malignant growth or tumour in the oropharynx
c. All of the above
d. I don’t know

17. What can cause oral cancer?

a. Tobacco use (smoking)
b. Alcohol use
c. Infection by the human papilloma virus (HPV)
d. All of the above
e. I don’t know

18. How can oral cancer (mouth cancer) be prevented?

a. Stop using tobacco (quit smoking).
b. Stop drinking alcohol.
c. Avoid excessive sun exposure to the lips
d. See the dentist regularly
e. All of the above
f. I don’t know

Section C: Attitude toward oral health

Please tick (√) one answer only for each statement

Statement		Strongly Disagree	Disagree	Neutral	Agree	Strongly agree
1.	I believe that only the dentist can prevent cavities	□	□	□	□	□
2.	I believe that brushing and flossing teeth can reduce the risk of tooth decay	□	□	□	□	□
3.	I believe that tooth loss before the age of 5 years is normal	□	□	□	□	□
4.	I believe that I am responsible for preventing the loss of my teeth	□	□	□	□	□
5.	I believe that we should brush our teeth at least twice per day	□	□	□	□	□
6.	If my gums bleed when I floss, this usually means that I am hurting my gums and I should stop flossing my teeth	□	□	□	□	□
7.	I believe that visiting the dentist is only necessary when I am experiencing pain	□	□	□	□	□
8.	I believe that teeth and gums should be cleaned every day	□	□	□	□	□
9.	I believe that fluoridated toothpaste is good for the teeth	□	□	□	□	□
10.	I believe that sugary foods and drinks should be avoided	□	□	□	□	□
11.	I believe that it is important to treat baby (primary) teeth	□	□	□	□	□
12.	I believe that regular dental visits are needed	□	□	□	□	□

Section D: Practice of oral health

Please tick (√) one answer only for each question.

1. **Does anyone assist you in brushing your teeth?**

   a. Yes
   b. No

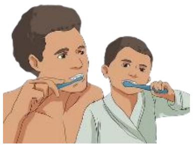

2. **How often do you brush your teeth?**
   a. Twice a day
   b. Once a day
   c. 2-3 times a week
   d. Less frequently
   e. Never

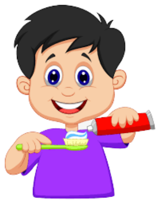

3. **Do you use toothpaste when you brush your teeth?**
   a. Yes
   b. No
   c. I never brush, so I never use toothpaste.
   d. I don't know

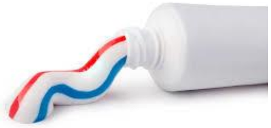

4. **Do you use toothpaste that contains fluoride?**
   a. Yes
   b. No
   c. I don't know

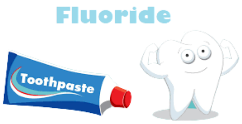

5. **How long do you spend brushing your teeth?**
   a. Less than 1 minute
   b. 1 minute
   c. 2 minutes
   d. I don't know

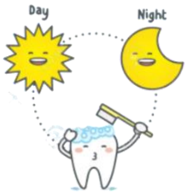

6. **How often do you clean between your teeth (using dental floss, a toothpick, or an interdental brush)?**
   a. Once a day or more
   b. 2-3 times a week
   c. Once a week
   d. Less frequently
   e. Never

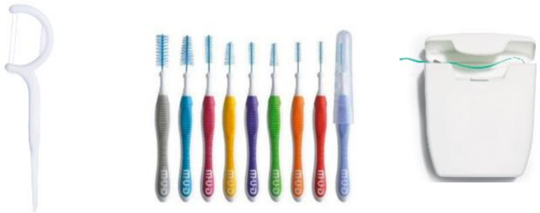

7. **How often do you replace your toothbrush?**
   a. Every 3 to 6 months
   b. Every 6 months
   c. Every 6-12 months
   d. Once a year
   e. When the toothbrush loses its bristles
   f. I don't know

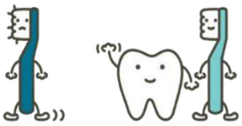

8. **How hard are the bristles on your toothbrush?**
    a. Hard bristles
    b. Soft bristles
    c. Medium bristles
    d. It doesn't matter
    e. I don't know

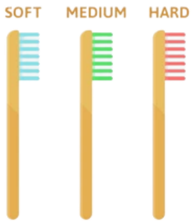

9. **Do you use <u>wooden toothpicks</u> to clean your teeth or gums?**
    a. Yes
    b. No
    c. I don't know

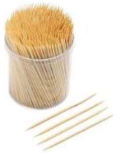

10. **Do you use <u>plastic toothpicks</u> to clean your teeth or gums?**
    a. Yes
    b. No
    c. I don't know

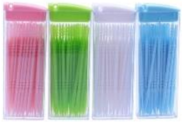

11. **Do you use <u>Miswak (chewstick)</u> to clean your teeth or gums?**
    a. Yes
    b. No
    c. I don't know

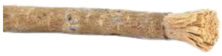

12. **Have you ever visited a dentist?**
    a. Yes, regularly
    b. Yes, but not regularly
    c. Never
    d. I can't remember

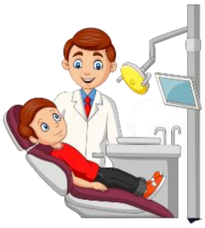

Supplement: Supplementary file 1 [file dentistry-14-00530-s001.zip › dentistry-4454937-supplementary.pdf]
